# Large language models for intelligent RDF knowledge graph construction: results from medical ontology mapping

**DOI:** 10.3389/frai.2025.1546179

**Published:** 2025-04-25

**Authors:** Apostolos Mavridis, Stergios Tegos, Christos Anastasiou, Maria Papoutsoglou, Georgios Meditskos

**Affiliations:** School of Informatics, Aristotle University of Thessaloniki, Thessaloniki, Greece

**Keywords:** LLM, ontology, knowledge graph, health data, RDF, SNOMED CT

## Abstract

The exponential growth of digital data, particularly in specialized domains like healthcare, necessitates advanced knowledge representation and integration techniques. RDF knowledge graphs offer a powerful solution, yet their creation and maintenance, especially for complex medical ontologies like Systematized Nomenclature of Medicine - Clinical Terms (SNOMED CT), remain challenging. Traditional methods often struggle with the scale, heterogeneity, and semantic complexity of medical data. This paper introduces a methodology leveraging the contextual understanding and reasoning capabilities of Large Language Models (LLMs) to automate and enhance medical ontology mapping for Resource Description Framework (RDF) knowledge graph construction. We conduct a comprehensive comparative analysis of six systems–GPT-4o, Claude 3.5 Sonnet v2, Gemini 1.5 Pro, Llama 3.3 70B, DeepSeek R1, and BERTMap—using a novel evaluation framework that combines quantitative metrics (precision, recall, and F1-score) with qualitative assessments of semantic accuracy. Our approach integrates a data preprocessing pipeline with an LLM-powered semantic mapping engine, utilizing BioBERT embeddings and ChromaDB vector database for efficient concept retrieval. Experimental results on a dataset of 108 medical terms demonstrate the superior performance of modern LLMs, particularly GPT-4o, achieving a precision of 93.75% and an F1-score of 96.26%. These findings highlight the potential of LLMs in bridging the gap between structured medical data and semantic knowledge representation, toward more accurate and interoperable medical knowledge graphs.

## 1 Introduction

The accelerating digitization of information across all sectors has created an unprecedented surge in the volume, velocity, and variety of data (Venkatachaliah, 2011; Chakraborty et al., 2017). This “data deluge” presents significant challenges for traditional data management and integration techniques, which often struggle to effectively handle the complexity and scale of modern datasets (Nashipudimath et al., 2020). This challenge becomes even more evident in fields such as healthcare, where vast amounts of data must be managed. The complexity arises from the diversity of formats, the nuanced interconnections within the data, and the presence of sensitive information. As a result, the demand for scalable, resilient, and semantically enriched methods for representing and integrating knowledge has never been greater.

The Resource Description Framework (RDF) knowledge graphs have emerged as a paradigm for addressing these challenges (Wu and Banerjee, 2014). RDF offers a flexible and expressive framework for representing interconnected data in a machine-readable format. By utilizing Uniform Resource Identifiers (URIs) to uniquely identify entities and defining relationships between them, RDF graphs enable the creation of semantically rich representations that facilitate advanced querying, reasoning and analysis (Zou, 2020). The visual nature of knowledge graphs further enhances their utility, providing intuitive means for exploring and interpreting datasets (Sayed Ahmed Soliman and Tabak, 2020). These characteristics make RDF knowledge graphs particularly suited for applications that require sophisticated data integration, such as drug discovery, personalized medicine, and clinical decision support systems.

Despite the advantages of RDF, several key challenges hinder the widespread adoption and effective utilization of knowledge graphs. The construction and maintenance of large-scale RDF graphs often require significant manual effort, particularly for ontology mapping and data integration (Singh et al., 2023). Conventional approaches to constructing knowledge graphs, including manual curation and rule-based techniques, often face challenges in scaling effectively to accommodate the continuous expansion of data. Additionally, interacting with RDF databases through SPARQL can be daunting for users unfamiliar with such technologies (Han et al., 2015). This difficulty reduces the accessibility of RDF knowledge graphs and hinders their broader practical adoption.

Mapping structured data, especially from common formats like CSV files, to RDF presents specific obstacles (Chaves-Fraga et al., 2020). Domain-specific terminology, abbreviations, and inconsistent data formats require significant preprocessing and interpretation to ensure accurate semantic representation within the RDF graph. Numeric data, prevalent in many datasets, needs careful contextualization and alignment with relevant ontologies, as raw numbers lack the inherent symbolic meaning crucial for RDF's semantic expressiveness. These challenges are particularly evident in the medical domain, where data is often highly structured but requires extensive domain knowledge for accurate mapping to established medical ontologies like SNOMED CT.

The recent advent of Large Language Models (LLMs) has revolutionized the field of natural language processing, offering promising new solutions for knowledge representation and semantic integration (Xue, 2024; Kulkarni, 2023). Trained on massive text corpora, LLMs like GPT-4o, Claude, and Gemini possess remarkable capabilities in understanding context, disambiguating terminology, and inferring relationships within text (Raiaan et al., 2024). Their ability to process and generate human-like text has opened up new possibilities for automating complex tasks, including knowledge graph construction, ontology mapping, and semantic enrichment (Kommineni et al., 2024; Trajanoska et al., 2023; Jia et al., 2024).

This paper introduces a novel methodology that harnesses the power of LLMs to address the persistent challenges in creating and utilizing RDF knowledge graphs, particularly in the context of medical ontology mapping. Our approach operates on two interconnected levels. First, we implement a robust data preprocessing pipeline that addresses the inherent heterogeneity and ambiguity of real-world medical data. This pipeline incorporates techniques such as terminology normalization, abbreviation expansion, and unit standardization, ensuring that the input data is consistent and amenable to semantic interpretation.

Second, we leverage the contextual understanding and reasoning capabilities of LLMs to perform intelligent ontology mapping. This involves formulating targeted prompts designed to elicit specific mappings between medical terms and concepts within the SNOMED CT ontology. To optimize retrieval and comparison of semantic representations, we employ a vector database (ChromaDB) populated with pre-computed BioBERT embeddings of both the input medical terms and the SNOMED CT concepts. This allows us to efficiently identify the most semantically similar concepts within the ontology based on cosine similarity between the embedding vectors. The integration of these two components—a robust data preprocessing pipeline and an LLM-powered semantic mapping engine—facilitates the automated generation of context-aware RDF triples, capturing the rich relationships embedded within the medical data and aligning them with the established semantic framework of SNOMED CT. This approach builds upon recent advancements in cloud-based RDF stores and distributed graph processing (Janke and Staab, 2018), enabling scalable and efficient knowledge graph construction. We specifically focus on the medical domain, demonstrating how LLMs, combined with a tailored preprocessing strategy and vector database integration, can be effectively employed to map complex medical terminology and structured data to the SNOMED CT ontology. This work aims to bridge the gap between structured data and semantic knowledge representation, facilitating the creation of more accurate, comprehensive, and interoperable medical knowledge graphs.

Our key contributions are threefold. First, we present a comprehensive comparative analysis of six distinct systems—GPT-4o, Claude 3.5 Sonnet v2, Gemini 1.5 Pro, Llama 3.3 70B, DeepSeek R1, and BERTMap—for medical ontology mapping and RDF knowledge graph construction. This analysis examines the advantages and drawbacks of each method, providing insights for researchers and practitioners looking to utilize LLMs in knowledge graph construction. Additionally, we propose a new evaluation framework for measuring the effectiveness of language models in mapping medical terminology. This framework integrates both quantitative metrics and qualitative evaluations of semantic accuracy, ensuring a comprehensive and rigorous assessment. Lastly, we showcase the enhanced capabilities of modern LLMs, particularly GPT-4o, in processing intricate medical concepts and relationships. Our findings indicate substantial advancements over the BERTMap baseline, with GPT-4o demonstrating a 44.91 percentage point improvement in precision (93.75% vs. 48.84%) and a 38.33 percentage point increase in F1-score (96.26% vs. 57.93%), highlighting the potential of LLMs to automated knowledge graph construction. This study highlights the impact of combining LLMs with RDF knowledge graphs to develop more intelligent, adaptive, and semantically enriched information systems. It paves the way for harnessing the vast and intricate realm of digital information to extract deeper insights and improve decision-making across multiple fields, which marks a crucial advancement in reshaping how we access, integrate, and manage information in complex settings (Lenzerini, 2018).

The remainder of this paper is structured as follows: Section 2 reviews related work in RDF knowledge graphs, ontology mapping, and the application of LLMs in semantic web technologies. Section 3 details our methodology, including the system architecture, testing framework, and evaluation metrics. Section 4 presents the experimental results, providing a detailed performance analysis of the evaluated systems. Finally, Section 5 discusses the findings, highlights the implications of our work, and outlines future research directions.

## 2 Related work

The Resource Description Framework (RDF) serves as a cornerstone of the Semantic Web, enabling structured data sharing on a global scale. As Berners-Lee (2019) explains, RDF extends the hypertext Web by using URIs to identify and describe resources, employing various serializations for data exchange. Its focus on data content meaning rather than structure makes it particularly suitable as a semantic data model for cloud computing (Kanmani et al., 2017). However, the growing size of RDF datasets presents significant challenges for data management systems, necessitating efficient storage techniques, indexing strategies, and query execution mechanisms (Wylot et al., 2018).

SPARQL, the standard query language for RDF, has motivated extensive theoretical studies of RDF and SPARQL fundamentals (Arenas et al., 2013). While RDF data can be handled using relational tables, querying large triple tables becomes computationally expensive due to the multiple nested joins required for graph queries (Wylot et al., 2018). The increasing availability of RDF data on the Web underscores the urgent need to address scalability issues in RDF data management (Arenas et al., 2013).

Knowledge graphs have emerged as powerful tools for representing complex, interconnected information in a machine-readable format. They are increasingly utilized across various domains, including libraries, digital humanities, and explainable machine learning (Haslhofer et al., 2018; Tiddi and Schlobach, 2022). These graphs represent concepts and their semantic relationships, supporting resource discovery, navigation, and visualization (Haslhofer et al., 2018). Recent research has focused on knowledge graph representation learning, acquisition, completion, and temporal aspects (Ji et al., 2022). Embedding methods have gained popularity, enabling various applications with implicit semantics derived from context (Kejriwal et al., 2019).

The process of generating RDF knowledge graphs from raw data has shifted from manual efforts to automated techniques. Recent approaches utilize existing ontologies, knowledge bases, and machine learning models to derive structured insights from various sources, including research publications (Constantopoulos and Pertsas, 2020). Advanced algorithms and tools, such as the SDM-RDFizer, have been designed to streamline the transformation of heterogeneous datasets into RDF representations (Iglesias et al., 2020). Additionally, real-time extraction of RDF triples from unstructured data streams has been explored through a combination of statistical analysis and machine learning strategies (Gerber et al., 2013).

Ontology mapping is essential for addressing heterogeneity challenges and ensuring semantic interoperability across diverse information sources (Benslimane et al., 2008). It plays a key role in knowledge graph construction by integrating multiple heterogeneous datasets (Iglesias-Molina et al., 2019). Various methods have been proposed, such as declarative mappings and language-independent templates using spreadsheets, which enhance maintainability and accessibility for non-experts (Iglesias-Molina et al., 2019). Additionally, frameworks, such as MapSDI, optimize semantic data integration through pre-processing based on mapping rules (Jozashoori and Vidal, 2019).

Furthermore, recent research has focused on enhancing the semantic interpretation of structured data sources in privacy-preserving environments. Karalka et al. (2023) propose SemCrypt, a framework for schema enrichment through semantic annotations and mappings to knowledge bases and ontologies, aiming to assess privacy-preserving technologies based on data sensitivity. This approach builds upon earlier work in semantic integration of heterogeneous data sources (Bergamaschi et al., 1999) and semi-automatic mapping of structured sources to ontologies (Knoblock et al., 2012), which facilitate the creation of shared ontologies, semantic relationships, and mapping rules for integrated data access. The importance of privacy preservation in data analysis is emphasized by Dwork (2011). By integrating semantic analysis with privacy-aware methodologies, researchers aim to create more sophisticated and intelligent strategies for managing sensitive data across domains, such as healthcare, finance and cyber threat intelligence.

The emergence of Large Language Models (LLMs) has significantly transformed natural language processing, showcasing exceptional performance in grasping context, meaning, and intricate relationships (Xue, 2024). Evolving from rule-based methods to highly advanced architectures, these models leverage transformer-based frameworks and sophisticated training paradigms to execute a wide range of tasks, including text generation, sentiment analysis and question answering (Kulkarni, 2023; Xue, 2024). Their capabilities extend well beyond artificial intelligence, shaping advancements in diverse fields, such as medicine, engineering, social sciences, and the humanities (Fan et al., 2024).

Recent studies use Machine Learning in different aspects of knowledge graph development and ontology alignment, demonstrating potential in areas, such as entity learning, ontology learning, and knowledge reasoning (Zhao et al., 2023). They have been applied to key tasks, including entity extraction, relation extraction, entity linking and link prediction (Zhao et al., 2023). Moreover, machine learning has been leveraged to automate data preparation and cleaning for knowledge graph curation, as well as data integration (Berti-Equille, 2019). Large Language Models (LLMs), in particular, show promise in streamlining data extraction and resolution processes for heterogeneous data sources (Remadi et al., 2024). For example, the LLMs4OM framework demonstrates the effectiveness of LLMs in ontology matching tasks, potentially surpassing traditional systems (Giglou et al., 2024). Additionally, integrating LLMs with scholarly knowledge graphs enhances query processing, enabling comprehensive and efficient information retrieval from academic research artifacts (Jia et al., 2024). In addition, transfer learning and pre-trained language models have significantly advanced natural language processing tasks, with domain-specific pre-training emerging as a powerful technique to enhance performance on specialized tasks (Zhong and Goodfellow, 2024). In this context, memory-augmented models have been proposed to address the potential loss of general knowledge during domain adaptation, combining domain-specific learning with preserved general knowledge (Wan et al., 2022).

Despite these advancements, several challenges remain in information extraction and query processing. Examples include handling ambiguity, scaling to large datasets and adapting to domain-specific terminology. Recently proposed solutions, such as systems to resolve query ambiguity using large-scale user behavioral data (Korayem et al., 2015), models for automatic term ambiguity detection (Baldwin et al., 2013), and query relaxation approaches, leverage external knowledge sources (Lei et al., 2020). These studies demonstrate progress, highlighting the need for advancements in handling ambiguity, scalability and domain adaptation in knowledge representation and retrieval systems.

While significant progress has been made in RDF knowledge graph construction, ontology mapping and the application of machine learning to these domains, the need for more sophisticated, context-aware approaches that can handle the complexity and nuance of real-world data remains. Our research is driven by this need and leverages recent advancements in LLMs for semantically rich RDF knowledge graphs.

## 3 Method

### 3.1 Implementation

**Figure 1:**
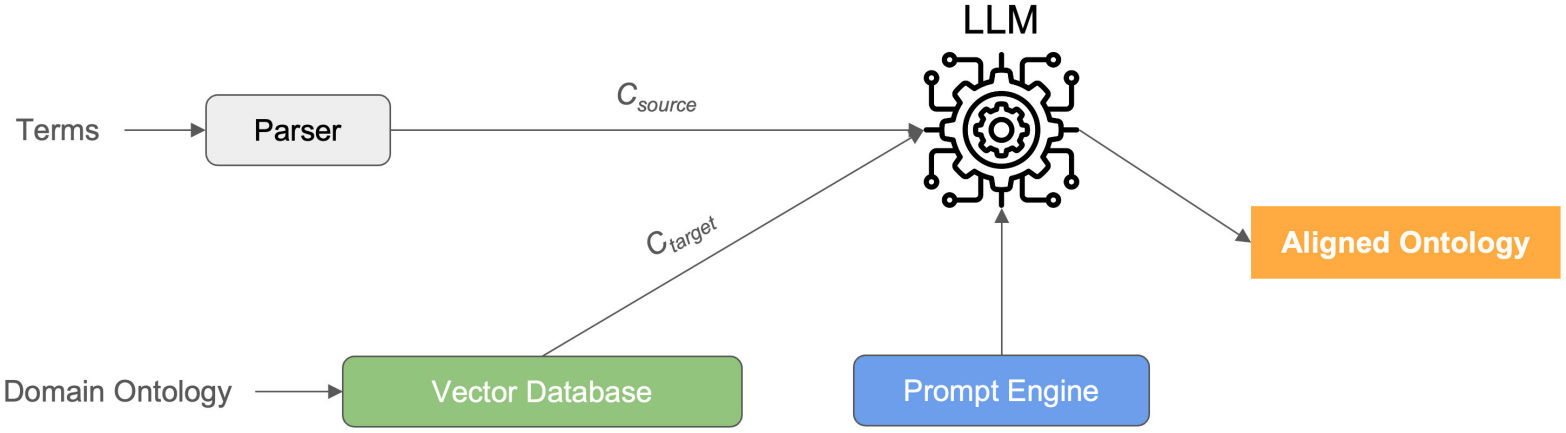
LLM-based system architecture.

Our study embarked on a meticulous comparative analysis to evaluate the efficacy of six distinct computational systems in the realm of medical ontology mapping and RDF knowledge graph construction (see Figure 1). This endeavor was motivated by the critical need for accurate and scalable methods to process the increasingly voluminous and complex data prevalent in modern healthcare settings. The systems under scrutiny were GPT-4o, Claude 3.5 Sonnet v2, Gemini 1.5 Pro, Llama 3.3 70B, DeepSeek R1 representing state-of-the-art Large Language Models (LLMs), and BERTMap, a well-established baseline system in ontology mapping. The central objective was to rigorously assess the potential of LLMs to surpass traditional methodologies in handling the intricacies of medical terminology, thereby facilitating the creation of semantically rich and accurate RDF knowledge graphs.

The selection of LLMs for this paper was based on several criteria, including their representation of both cutting-edge and open-source models, their proven effectiveness in handling complex tasks, and their accessibility for ensuring reproducibility. Among the state-of-the-art models, we included GPT-4o, Claude 3.5 Sonnet v2, and Gemini 1.5 Pro, developed by OpenAI, Anthropic, and Google, respectively. These models represent the forefront of LLM advancements, each with distinct architectural strategies and strong performance in complex reasoning and domain-specific applications, while their stable API access facilitates experimental reproducibility. To provide a more comprehensive analysis and acknowledge the increasing significance of open-source alternatives in healthcare, we incorporated two high-performing open-source LLMs: Llama 3.3 70B from Meta, which boasts 70 billion parameters and excels across diverse tasks, and DeepSeek R1, a recently introduced open-source model demonstrating promising capabilities in language understanding and generation. BERTMap was selected as the baseline due to its well-established role in ontology mapping research and its widespread use as a benchmark system.

Implementation parameters:

- GPT-4o: Temperature = 0.1, max tokens = 2,048, cost = $0.03/1K tokens.
- Claude 3.5 Sonnet v2: Temperature = 0.1, max tokens = 2048, cost = $0.015/1K tokens.
- Gemini 1.5 Pro: Temperature = 0.1, max tokens = 2,048, cost = $0.01/1K tokens.
- Llama 3.3 70B: Temperature = 0.1, max tokens = 2,048, indirect costs for setup.
- DeepSeek R1: Temperature = 0.1, max tokens = 2,048, indirect costs for setup.
- BERTMap: Default parameters as per public implementation.

The proposed framework in this paper is a key component of the ENCRYPT project,^1^ which focuses on addressing privacy and security challenges in critical sectors such as healthcare, finance, and entertainment. Within the ENCRYPT framework, Knowledge Graphs (KGs) play a central role in enabling data interoperability, semantic understanding, and efficient information sharing across heterogeneous datasets. This work presents a comprehensive approach to constructing semantically rich RDF Knowledge Graphs specifically tailored for healthcare data. By leveraging advanced privacy-preserving technologies such as Fully Homomorphic Encryption (FHE), Secure Multi-Party Computation (SMPC), and Differential Privacy (DP), the ENCRYPT project ensures that sensitive data can be securely managed and analyzed in compliance with GDPR regulations. Our approach integrates semantic web standards, utilizing ontologies such as SNOMED CT and DICOM to enable accurate semantic representation of medical data. This paper highlights the full methodology, combining LLM-powered ontology mapping, a robust preprocessing pipeline, and vector database integration, to demonstrate the transformative potential of Knowledge Graphs in addressing the complexities of healthcare data management.

In constructing our evaluation pipeline, a critical component was the selection and implementation of a vector database to efficiently manage and retrieve the embeddings representing our medical terms (*C*_source_) and the corresponding concepts within the SNOMED CT ontology (*C*_target_). For this purpose, we employed ChromaDB, an open-source vector database renowned for its ease of use, scalability, and seamless integration with natural language processing workflows. ChromaDB served as the central repository for storing and indexing the vector representations generated for both the input medical terms and the SNOMED CT concepts.

To generate these vector representations, we leveraged pre-trained embedding models tailored for biomedical text. Specifically, we utilized the BioBERT model, a domain-specific variant of BERT trained on a large corpus of biomedical literature. BioBERT has demonstrated superior performance in capturing the semantic nuances of medical terminology compared to general-purpose embedding models. Each medical term in our evaluation dataset and each concept in the relevant subset of SNOMED CT were processed through BioBERT to produce dense vector embeddings. These embeddings encapsulated the semantic meaning of each term or concept, allowing for efficient similarity comparisons within the vector space.

For the Large Language Models (LLMs)—GPT-4o, Claude 3.5 Sonnet v2, Gemini 1.5 Pro, Llama 3.3 70B, and DeepSeek R1—we interacted with them via their respective API endpoints. The API calls were programmatically managed using Python, facilitating seamless integration with our evaluation framework. The prompts presented to the LLMs were carefully engineered to guide them toward generating mappings to SNOMED CT concepts. Specifically, we employed a structured prompt formatter (prompt engine) that included the medical term to be mapped, a clear instruction to provide the corresponding SNOMED CT identifier, and a request to provide a confidence score for the proposed mapping. This structured approach ensured consistency in our interactions with the LLMs and facilitated the extraction of the relevant information from their responses.

The responses obtained from the LLMs were then parsed programmatically to extract the proposed SNOMED CT identifiers and confidence scores. To identify the most relevant concepts, we performed a nearest neighbor search within the ChromaDB vector store. The vector embedding of the input medical term, as generated by BioBERT, was used as the query vector. The distance metric employed for the nearest neighbor search was cosine similarity, which measures the cosine of the angle between two vectors, providing a robust indicator of semantic similarity. The top-k nearest neighbors, as determined by cosine similarity, were retrieved from ChromaDB, representing the SNOMED CT concepts most semantically similar to the input medical term.

For the baseline system, BERTMap, we utilized the publicly available implementation and adhered to the recommended parameter settings. BERTMap operates by generating contextualized word embeddings for both the source and target ontologies (in our case, the input medical terms and SNOMED CT) and subsequently computes a similarity matrix based on cosine similarity. Alignment candidates are then identified through a greedy matching algorithm.

To ensure the reproducibility of our results and to manage the complexities of our experimental setup, we employed a containerization approach using Docker. Each component of our pipeline, including the vector database, the embedding generation scripts, the LLM interaction modules and the evaluation scripts, was encapsulated within a Docker container. This strategy ensured consistency across different environments and streamlined the deployment of our evaluation framework. Additionally, we employed a robust version control system via Git to systematically track code modifications, experimental parameters, and evaluation outcomes. This approach ensured detailed documentation of our methodology and streamlined collaboration among the researchers involved. Beyond the core technological aspects, we implemented thorough data preprocessing procedures to maintain the quality and consistency of input data. These steps encompassed standardizing the formatting of medical terms, managing abbreviations and acronyms, and resolving terminology inconsistencies. All preprocessing procedures were carefully documented to enhance transparency and reproducibility.

Throughout the evaluation process, we monitored the performance of our system, paying close attention to computational resource utilization, processing times and potential bottlenecks. This performance monitoring allowed us to identify areas for optimization and to ensure the scalability of our approach. The datasets and prompts used for this study can be accessed at the GitHub repository.^2^

### 3.2 Evaluation

To conduct a robust and representative evaluation, we assembled a comprehensive dataset comprising 108 distinct medical terms. This dataset was meticulously curated to reflect the diverse spectrum of clinical information routinely encountered in healthcare, encompassing patient demographics, physiological measurements, disease classifications, therapeutic interventions, and intricate diagnostic and procedural terms. This diversity was paramount to ensuring that the evaluation probed the full breadth of the systems' capabilities, challenging them with a range of linguistic complexities from straightforward measurements like age and weight to nuanced concepts such as disease staging and treatment withdrawal. The selection of these terms was informed by extensive consultation with domain experts, ensuring their relevance and representativeness within the medical field.

Prior to the computational analysis, we established a definitive ground truth to serve as the benchmark for evaluating the performance of the six systems. This was achieved through a rigorous expert elicitation process involving a panel of seasoned medical professionals and ontology engineers. Each expert independently reviewed the dataset, proposing mappings for each medical term to corresponding concepts within the SNOMED CT ontology. The selection of SNOMED CT, a globally recognized comprehensive medical ontology, was deliberate, given its extensive coverage of clinical concepts and its pivotal role in enabling interoperability in healthcare information systems. Following the individual review, a collaborative session was convened wherein the experts discussed discrepancies, negotiated disagreements, and ultimately reached a consensus on the most appropriate mappings for each term. This meticulous consensus-building process was particularly vital for terms exhibiting ambiguity or multiple valid interpretations, reflecting the complex and multifaceted nature of medical language. The resultant expert-validated ground truth provided a reliable standard against which the computational systems' performance could be measured.

While medical ontologies, like SNOMED CT, involve nuanced relationships, temporal data, and hierarchical structures, the dataset used in our study focused specifically on real-world terms extracted from patient data within a clinical setting. This focus inherently simplified the mapping task. The most frequent relationships were those directly associated with a patient (e.g., “patient has condition X”). The temporal scope primarily involved the patient's current condition and history, limiting the complexity of temporal relationships. Additionally, the terms used often focused on specific diagnoses, procedures, and medications, resulting in a relatively flattened hierarchy for the mapping task. In essence, the dataset reflected a practical use case where the complexities of SNOMED CT were naturally constrained by the context of patient data. This allowed us to focus on evaluating the models' ability to accurately map real-world medical terms to SNOMED CT concepts within this specific domain.

With the ground truth established, we proceeded to systematically evaluate the six computational systems under controlled conditions. Each of the 108 medical terms was presented as input to GPT-4o, Claude 3.5 Sonnet v2, Gemini 1.5 Pro, Llama 3.3 70B, DeepSeek R1, and BERTMap. For the LLMs, we employed their default model configurations without any task-specific fine-tuning, simulating a real-world scenario where researchers might utilize these models out-of-the-box. The prompts provided to these models were carefully designed to elicit mappings to SNOMED CT concepts, framed in a clear and unambiguous manner. BERTMap, serving as the baseline, was executed using its standard parameter settings, representing the typical usage of this system within the ontology mapping community. To eliminate potential bias during the evaluation process, we implemented a blind validation protocol. The mappings generated by each system were presented to the evaluators without disclosing the originating system, thereby preventing any subconscious influence on the judgment of the results' quality.

The quantitative evaluation of the systems' performance was based on standard classification metrics: precision, recall, and F1-score. These metrics were derived from a comprehensive confusion matrix, categorizing each mapping outcome as a true positive (TP), false positive (FP), false negative (FN), or true negative (TN). Precision measured the proportion of correctly identified mappings out of all mappings proposed by a system, while recall assessed the proportion of correctly identified mappings out of all possible correct mappings in the ground truth. The F1-score (the harmonic mean of precision and recall) provided a balanced measure of the system's overall accuracy. Beyond these quantitative metrics, we have also performed a qualitative analysis to assess the semantic relevance, clinical appropriateness and contextual accuracy of the generated mappings. This analysis had an important role in assessing the systems' capability to process complex medical concepts, including temporal relationships (e.g., symptom onset), detailed anatomical descriptions and multi-step procedural terms. Domain experts conducted the review, examining the mappings not only for their technical accuracy but also for their clinical relevance and meaningfulness within the medical context.

Furthermore, we applied a chi-square test of independence to determine whether the performance differences among the six systems were statistically significant. This test allowed us to determine whether the distribution of TP, FP, FN, and TN across the systems deviated significantly from what would be expected by chance. To quantify the magnitude of any detected associations, Cramer's V was calculated as a measure of effect size. This analytical approach that combines quantitative metrics, qualitative evaluation and statistical testing, provided a robust assessment of the performance of GPT-4o, Claude 3.5 Sonnet v2, Gemini 1.5 Pro, Llama 3.3 70B, DeepSeek R1, and BERTMap in the task of medical ontology mapping.

Furthermore, we documented the specific challenges encountered by each system, including recurring error patterns and areas of ambiguity. This error analysis provided useful insights into the inherent strengths and limitations of each approach. The transparency and thoroughness of our methodology, from dataset construction to evaluation, were designed to ensure the reproducibility of our findings and to provide a solid foundation for subsequent advancements in the field of automated medical knowledge representation and semantic integration.

## 4 Results

**Figure 2:**
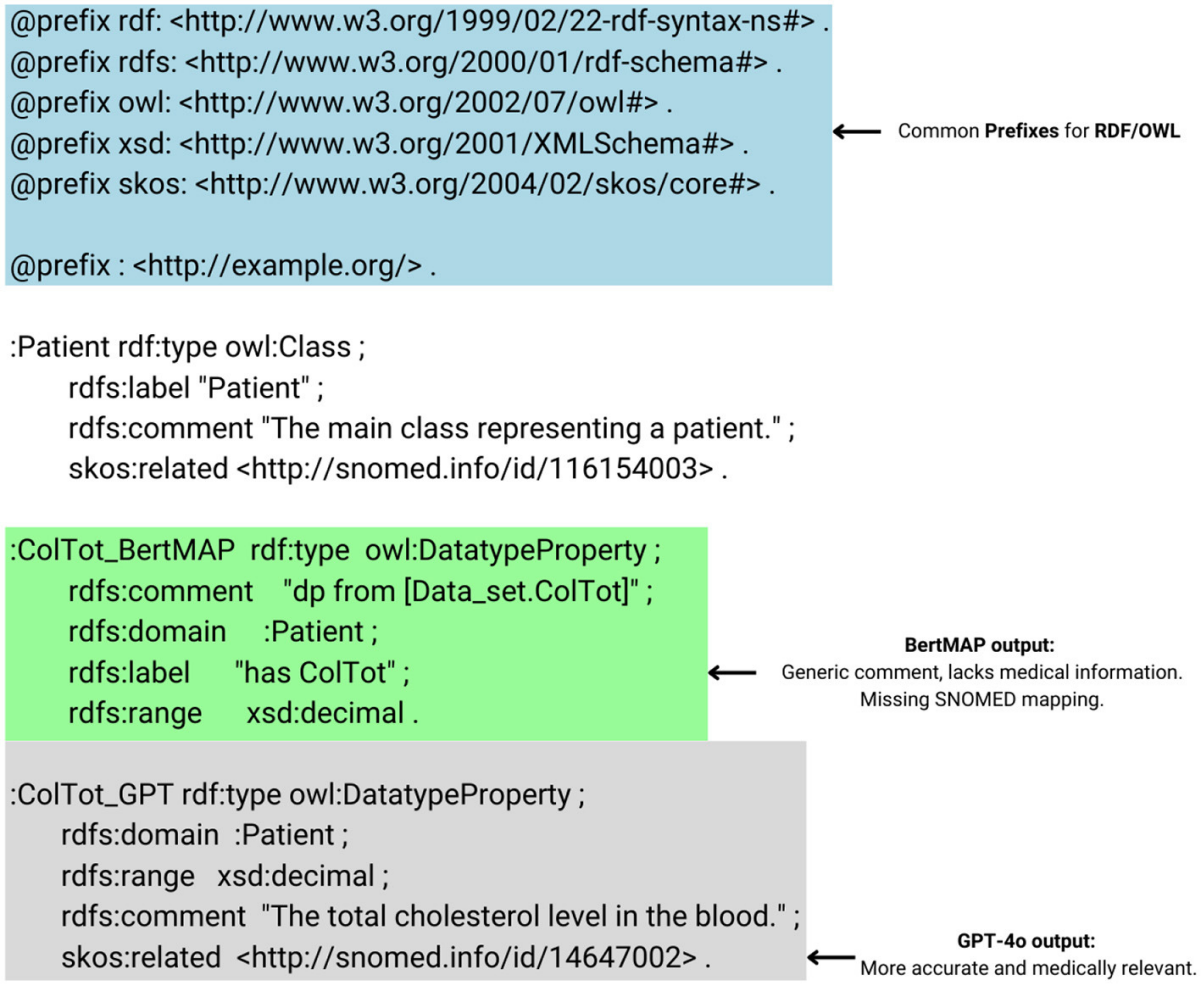
A comparison between the results of the baseline method and the top performance LLM.

Our evaluation comparing the LLMs and BERTMap revealed substantial differences in performance across multiple metrics. Analysis of the 108 medical terms in our dataset demonstrated varying levels of effectiveness among the systems, with GPT-4o showing superior overall performance. Figure 2 depicts an example of a comparison between the results of the baseline method and the top performance LLM.

GPT-4o achieved the highest precision (93.75%) among all systems, significantly outperforming Gemini 1.5 Pro (60.27%), Claude 3.5 Sonnet v2 (53.75%), BERTMap (48.84%), DeepSeek R1 (25.76%), and Llama 3.370B (19.19%). In terms of recall, GPT-4o led with 98.90%, followed by Llama 3.370B (70.37%), BERTMap (71.19%), Claude 3.5 Sonnet v2 (69.35%), Gemini 1.5 Pro (66.67%), and DeepSeek R1 (32.69%). The combined effect of these metrics resulted in F1-scores of 96.26% for GPT-4o, 63.31% for Gemini 1.5 Pro, 60.56% for Claude 3.5 Sonnet v2, 57.93% for BERTMap, 30.16% for Llama 3.370B, and 28.81% for DeepSeek R1, demonstrating GPT-4o's substantial advantage in overall performance (see Table 1).

**Table 1:** Comparison of method performance metrics.

**Method**	**Precision**	**Recall**	**F1-score**
GPT-4o	93.75	98.90	96.26
Claude 3.5 Sonnet v2	53.75	69.35	60.56
Gemini 1.5 Pro	60.27	66.67	63.31
BERTMap	48.84	71.19	57.93
Llama 3.3 70B	19.19	70.37	30.16
DeepSeek R1	25.76	32.69	28.81

Detailed analysis of the confusion matrix metrics revealed significant differences among the systems (Table 2). GPT-4o demonstrated the best performance, generating 90 true positives, 6 false positives, 1 false negative, and 11 true negatives. Gemini 1.5 Pro produced 44 true positives, 29 false positives, 22 false negatives, and 13 true negatives, while Claude 3.5 Sonnet v2 achieved 43 true positives, 37 false positives, 19 false negatives, and 9 true negatives. BERTMap produced 42 true positives, 44 false positives, 17 false negatives, and 5 true negatives. Llama 3.3 70B generated 19 true positives, 80 false positives, 8 false negatives, and 1 true negatives, while DeepSeek R1 produced 17 true positives, 49 false positives, 35 false negatives, and 7 true negatives. The notably lower number of false positives and false negatives in the GPT-4o system (6 and 1, respectively) compared to the other systems indicates superior discrimination ability in mapping medical terminology.

**Table 2:** Contingency table.

**Method**	**True positives**	**False positives**	**True negatives**	**False negatives**
GPT-4o	90	6	11	1
Claude 3.5 Sonnet v2	43	37	9	19
Gemini 1.5 Pro Latest	44	29	13	22
BERTMap	42	44	5	17
Llama 3.3 70B	19	80	1	8
DeepSeek R1	17	49	7	35

**Figure 3:**
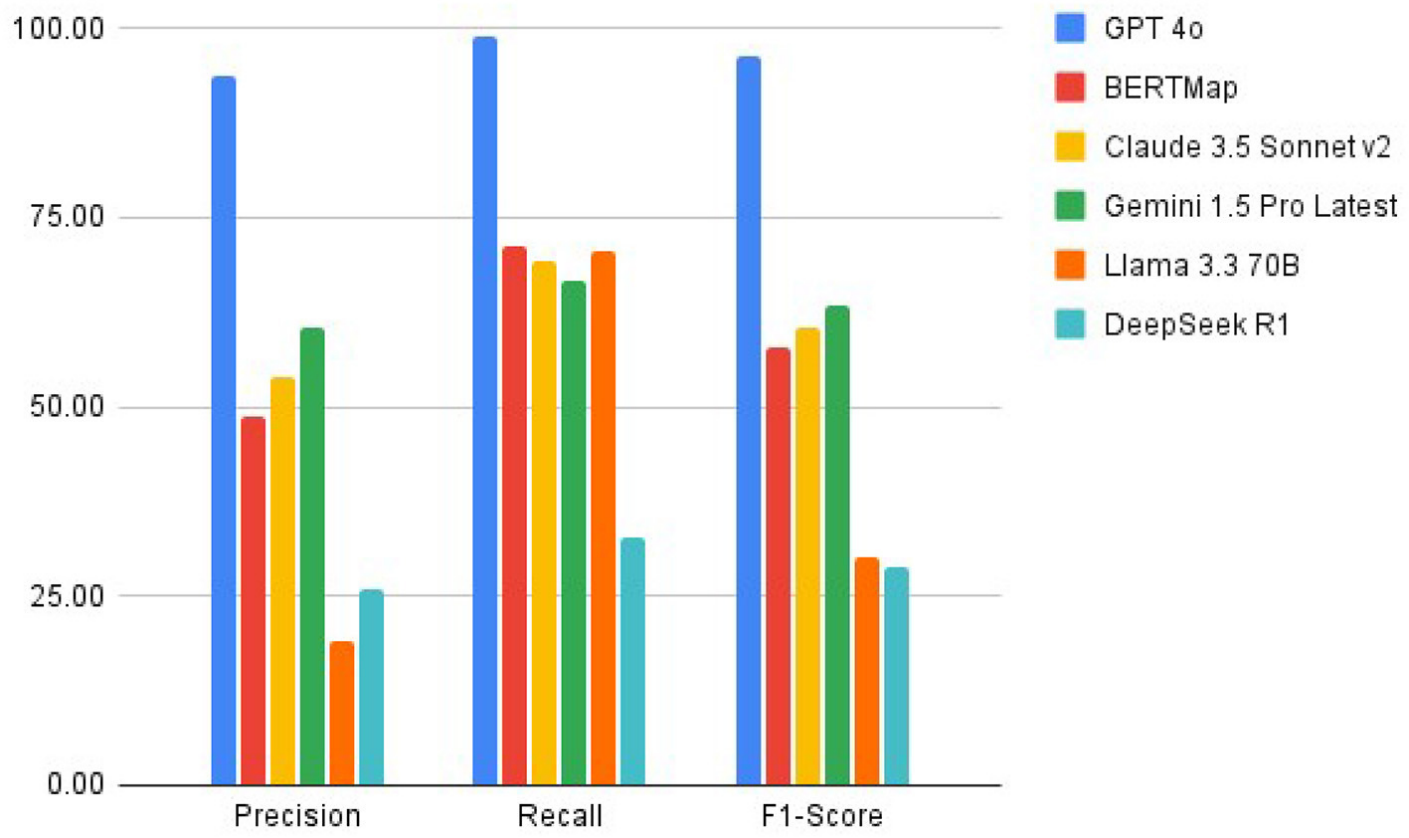
A depiction of the performance for all methods.

Performance analysis across different categories of medical terms revealed specific patterns (see Figure 3). All systems performed relatively well with basic clinical measurements such as sex, age, weight, height, and BMI. However, GPT-4o demonstrated superior performance in mapping complex medical concepts, particularly in areas of diagnostic procedures and examinations, treatment-related terminology, temporal relationships, and disease complications and classifications. Llama 3.3 70B and DeepSeek R1 showed particular weakness in handling these more complex concepts, with high rates of incorrect mappings.

The systems' performance diverged most notably in handling complex procedural terminology and multi-component medical concepts. GPT-4o maintained high accuracy in these cases, while the other systems showed increased error rates. For instance, GPT-4o accurately mapped complex terms such as “Exam Type,” “Diagnostic question,” and “withdrawal of therapy,” where the other systems produced more inconsistent results.

Error analysis revealed that GPT-4o's few misclassifications were primarily concentrated in specific areas of complex quantitative measurements, multi-parameter clinical assessments, and certain anatomical treatment locations. These included terms such as “min_ECGstress,” “ECGstress_Result,” and anatomical treatment specifications. While all systems demonstrated competence in basic medical terminology mapping, GPT-4o offered substantial improvements in handling complex, context-dependent medical concepts and relationships.

A chi-square test of independence was performed to examine the relationship between the six ontology matching methods (GPT-4o, Claude 3.5 Sonnet v2, Gemini 1.5 Pro Latest, Llama 3.3 70B, DeepSeek R1, and BERTMap) and their performance outcomes (TP, FP, TN, and FN; see Table 1). The test revealed a statistically significant difference between the methods, χ^2^ (15, *N* = 648) = 196.94, *p* < 0.001, Cramer's V = 0.32 (indicating a large effect size).

*Post-hoc* examination of the standardized residuals indicates that GPT-4o demonstrated significantly higher true positive rates and lower false positive rates than expected under the null hypothesis. Specifically, GPT-4o achieved 90 true positives compared to 17–44 for the other methods, representing a substantial performance advantage.

## 5 Discussion

The results of our comparative study demonstrate significant variations in performance across six systems evaluated for medical ontology mapping and RDF knowledge graph creation. GPT-4o's superior performance, with precision of 93.75%, stands in stark contrast to Gemini 1.5 Pro (60.27%), Claude 3.5 Sonnet v2 (53.75%), BERTMap (48.84%), DeepSeek R1 (25.76%), and Llama 3.370B (19.19%). This wide performance gap illustrates the substantial advancement in automated mapping accuracy achieved by the most advanced LLMs, while also highlighting the challenges faced by smaller or less sophisticated models.

The performance hierarchy among the systems provides valuable insights into the evolution of language models for specialized tasks. GPT-4o's exceptional performance suggests that its architecture and training approach are particularly well-suited for handling medical terminology. The significant drop in performance across other models creates a clear stratification: Gemini 1.5 Pro's intermediate performance (F1-score 63.31%) positions it as the second-best option, followed by Claude 3.5 Sonnet v2 (60.56%) and BERTMap (57.93%), with DeepSeek R1 (28.81%) and Llama 3.370B (30.16%) showing substantially lower capabilities.

Of particular interest is Llama 3.370B's relatively high recall (70.37%) despite its low precision (19.19%), suggesting a tendency toward over-generation of mappings. In contrast, DeepSeek R1's more balanced but lower precision (25.76%) and recall (32.69%) indicate consistent but limited capabilities across all aspects of the task.

All systems demonstrated basic competence in mapping simple clinical measurements, but their performance diverged significantly when handling complex medical concepts. GPT-4o maintained high accuracy across sophisticated scenarios, including disease terms, temporal relationships, and procedural terminology. The progressive degradation in performance as concept complexity increased was most pronounced in the open source models like Llama 3.370B and DeepSeek R1, which struggled particularly with nuanced medical terminology.

Error analysis reveals distinct patterns across the systems. GPT-4o's minimal error profile (6 false positives and 1 false negative) stands in sharp contrast to the higher error rates of other systems. Gemini 1.5 Pro and Claude 3.5 Sonnet v2 showed similar error patterns, with moderate false positive and false negative rates, while BERTMap exhibited a moderate false positive rate (44). The high false positive rates in Llama 3.370B and DeepSeek R1 suggest that smaller models struggle significantly with discriminating valid mappings from invalid ones, highlighting the importance of model scale and training data quality in achieving reliable performance.

We certainly acknowledge limitations across all systems. Performance variability depends on training data quality and coverage, with rare or highly specialized concepts presenting challenges for all methods. GPT-4o's few misclassifications centered on complex quantitative measurements and multi-parameter clinical assessments, while other systems showed broader patterns of error across multiple concept types. The need for domain expert validation remains, particularly for critical applications, though the level of required oversight varies significantly among the systems.

A possible reason for GPT-4o's struggle with complex quantitative measurements and multi-parameter clinical assessments lies in the inherent challenges of such data. Quantitative medical data often require precise numerical reasoning and context-specific interpretation, which can be difficult for language models primarily trained on textual data. Multi-parameter clinical evaluation involves intricate relationships among numerous variables, requiring not only precise recognition of individual terms but also the ability to synthesize and contextualize multiple data points simultaneously. While GPT-4o demonstrated the highest overall performance, the few misclassifications were mainly relevant to these more complex areas, suggesting that the strengths in language comprehension do not fully extend to the complex quantitative reasoning that is necessary for multi-parameter clinical assessments. This underlines the need for further improvements in numerical reasoning capabilities in LLMs, particularly in highly specialized domains, such as healthcare.

Future research presents several promising directions. The significant performance gap we observed between GPT-4o and smaller models, like Llama 3.3 70B and DeepSeek R1, indicates that architectural optimizations and refined training strategies could enhance the capabilities of more compact models. A major goal here is to develop more efficient architectures that can match GPT-4o's performance, while requiring fewer computational resources. These insights have practical implications. Although GPT-4o excels in medical terminology mapping, its high computational demands and cost may limit its feasibility for certain applications. The varying performance characteristics across models emphasize the necessity of balancing accuracy, efficiency and resource constraints when selecting a system for specific use cases.

Finally, the dataset of 108 medical terms, derived from real-world usage in a public hospital, was curated to reflect the practical challenges of medical language in clinical settings. A key advantage of this dataset is the availability of expert-validated ground truth, ensuring a reliable standard for evaluating LLMs, an aspect often difficult to achieve with larger datasets. While a broader dataset could enhance generalizability, the focus here was on assessing model performance in a realistic scenario with high-quality annotations. The selected terms span diverse clinical information, including patient demographics, physiological measurements, disease classifications, and treatments, providing a representative sample for medical ontology mapping. Additionally, qualitative analysis by domain experts offers critical insights into handling complex medical concepts. Future work aims to expand this evaluation with a larger dataset covering more medical subfields and data formats to further test the models' scalability and adaptability.

## 6 Conclusions

Our research highlights the evolving role of different LLM architectures in advancing RDF knowledge graphs. By comparing multiple systems, we've demonstrated not only the current state of the art but also the progression of capabilities in this field. The superior performance of GPT-4o suggests a pathway toward more intelligent, adaptive, and semantically rich knowledge representation systems, while the varying capabilities of other systems provide insights into alternative approaches and potential areas for improvement.

Our comparative study reveals a clear hierarchy in current LLM capabilities for knowledge graph creation and ontology mapping, with performance ranging from GPT-4o's exceptional results, even without fine-tuning, to the more limited capabilities of smaller models like Llama 3.370B and DeepSeek R1. This spectrum of performance highlights both the remarkable progress in the field and the continuing challenges in developing more efficient and accessible solutions. As these technologies evolve, we anticipate further improvements across all systems, potentially narrowing the current performance gaps while maintaining high standards of accuracy and reliability. Future work will explore the models' performance with datasets that involve more diverse relationships, temporal data, and hierarchical structures. Also the investigation of hybrid approaches that combine symbolic reasoning with machine learning, as well as the performance of different LLMs in niche areas of medical terminology mapping, especially for contexts with limited computational resources, would significantly expand the scope of this work.

## Data Availability

The original contributions presented in the study are included in the article/supplementary material, further inquiries can be directed to the corresponding author.

## Footnotes

^1^ https://encrypt-project.eu/

^2^ https://github.com/enchatted/llms-kgs
